# Symptoms Resulting from Anæsthesia by Ether in Young Subjects

**Published:** 1877-06

**Authors:** 


*Symptoms Resulting from Anæsthesia by Ether in Young Subjects.*—Dr. Leon Tripier read a paper on this subject before the French Association for the Advancement of Science. He related three cases in which the administration of ether for surgical operations was attended by an arrest of respiration, and though, after long efforts at restoration recoveries took place, the patients were placed in a most alarming condition. He also instituted experiments on young cats with ether, and found, as in young human subjects, an arrest of respiration often occured. Older animals were less liable to this accident. He, therefore, considers anæsthesia by ether in young subjects as dangerous, and that chloroform for them should be always preferred.

